# Integrating expert patients perspectives on the processes of engaging them in early medical education

**DOI:** 10.1186/s40900-024-00655-2

**Published:** 2024-11-19

**Authors:** Jaimy Saif, Duane Mellor, David Rogers, Claire Stocker

**Affiliations:** 1 Aston Medical School, College of Health and Life Sciences, Aston University, Birmingham, UK; 2 Expert by experience, Birmingham, UK

**Keywords:** Expert patients, Person-centred medical education

## Abstract

In the practice of healthcare, a new paradigm has emerged that perhaps challenges traditional notions of patient-clinician relationships. This shift involves recognising the invaluable role of expert patients, individuals who possess first-hand experience of life with their specific health conditions or chronic illnesses. These individuals have gained deep practical insights, knowledge, and coping strategies through their personal journeys. Modern healthcare practice focusing on individualised care necessitates that patients and their experiences become integral to the education of future healthcare professionals, from the start of their learning journeys, steering us toward more compassionate and person-centred approaches. This communication article underscores the importance of developing structured, coordinated programs that involve patients in curriculum design, implementation, and evaluation. By fostering authentic partnerships, medical education can create richer learning environments that promote compassionate care and better prepare future healthcare professionals. Ultimately, embracing patient perspectives as integral to the educational process is essential for improving healthcare delivery and outcomes.

## Abstract (plain language summary)

This comment article examines the evaluation of expert patients—individuals with personal health condition experiences—on their involvement in early years medical education. Recently, the amount of early clinical exposure provided by UK medical schools has fallen, with medical education institutions urged to consider alternative ways, such as expert patients to impart this experience to early years medical students. The purpose of incorporating expert patients’ feedback is to optimise their involvement by addressing their opinions on session formats, accessibility, and clarity, thereby enhancing the learning experience for both students and patients. This involvement benefits patients by boosting their self-confidence, satisfaction, and understanding of their conditions, while allowing them to contribute meaningfully to the healthcare system. For medical students, it enhances communication skills, empathy, and understanding of patient perspectives. Co-created by expert patients from the Silver Lining Brain Injury Charity and academics from Aston Medical School, the article presents feedback aimed at refining Expert Patient (EP) sessions. It also addresses challenges like inconsistent terminology and lack of standardised assessments and calls for institution-wide programs that integrate patient perspectives throughout medical education. The article emphasises the need for ongoing collaboration to ensure that patient voices shape educational experiences, thereby enhancing person-centered care and improving healthcare outcomes.

## Background

‘*To study the phenomenon of disease without books is to sail an uncharted sea*,* while to study books without patients is not to go to sea at all.’-* Sir William Osler.

Programs that incorporate patients as educators and instructors of clinical skills emerged in the early 1970s, stemming from the concept of the ‘programmed patient’ developed by Barrow and Abrahamson, using actors to portray patient encounters in medical education in USA [1]. These programmes were created to address challenges faced by doctors in effectively teaching, imparting, and developing communication and clinical skills in medical students. The concept of the ‘programmed patient’ involved utilising real patients as instructors in simulated or controlled settings to provide students with hands-on experience and practice [1]. By directly involving patients in the teaching process, medical educators aimed to bridge the gap between theoretical knowledge and practical application of clinical skills. This was built on the idea that patients possess unique insights and perspectives that can greatly enhance the learning experience for medical students [2, 3]. Patients, through their own lived experiences, can offer valuable feedback, guidance, and first-hand accounts of various medical conditions and scenarios [4]. This person-centred approach to teaching clinical skills provided students with a more realistic and immersive learning environment [5]. Over the past decade, in the United Kingdom healthcare policy makers and professional regulators have mandated the involvement of patients and the public in all aspects of healthcare [6]. The Department of Health document *Equity and Excellence*: *Liberating the NHS*12 emphasised the need for bringing patients back into the centre of all health care: “Nothing about me without me” [7] which explicitly refers to healthcare decisions relating to their care. The General Medical Council (GMC) emphasise importance of explicit inclusion of patient and public involvement in all aspects of medical education [8]. Interestingly, patient and public involvement in education is also mandated by other Healthcare Professional Statutory Regulated Bodies (PSRBs) such as General Pharmaceutical Council [9] and Nursing and Midwifery Council [10]. Collectively, these calls have led to a shift in the role of patients in healthcare education, evolving from being passive resources to becoming active partners involved in wide spectrum including design, implementation, and assessment of medical education [11, 12].

Engaging with real patients offers a plethora of benefits to students, including perceived relevance to their studies, an enriched understanding of patient perspectives, and improved communication skills [3, 13]. Additionally, students experience increased confidence when interacting with patients, especially during physical and especially intimate examinations, in a non-threatening environment facilitated by patient involvement [14, 15]. Comparisons of student perceptions before and after engaging with patients reveal positive changes in attitudes and assumptions about the role of patients in the doctor-patient relationship [16]. This results with students becoming more empathetic, compassionate and sensitive to the needs of vulnerable populations, such as those with chronic illnesses, disabled children, mental health conditions, and the elderly [17, 18]. Although independent verification of these perceptions is yet to be explored, students have been shown to learn physical examination skills equally well from patient teachers as they do from physicians [19], reinforcing the importance of patient involvement in shaping future healthcare professionals.

### The empowering effects of patient involvement in medical education: benefits for expert patients

Evaluating the impact of patient interaction on the patients involved is of equal importance. Previous studies have highlighted several benefits experienced by expert patients who participate in medical education initiatives. Firstly, engaging as expert patients leads to a better understanding of their own health conditions [20]. By sharing their experiences and knowledge with medical students, these patients gain insights into their conditions and treatment options, ultimately empowering them to take a more active role in managing their own health [20]. Secondly, patient involvement contributes to a deeper understanding of health services and doctor-patient relationships. Through their interactions with medical students, expert patients gain knowledge about healthcare systems, medical practices, and the dynamics of doctor-patient interactions. This increased understanding enables them to navigate healthcare services more effectively and engage in more meaningful and collaborative relationships with healthcare providers [21]. Moreover, personal satisfaction and empowerment are reported outcomes of patient involvement [21]. By actively contributing to medical education, expert patients feel valued and appreciated, which in turn enhances their sense of empowerment and satisfaction [22]. They become active participants in shaping the education and training of future healthcare professionals.

Increased confidence is another benefit observed among expert patients involved in medical education [17]. By sharing their experiences and expertise, these patients develop a sense of confidence in their knowledge and ability to contribute meaningfully to the learning process. This enhanced confidence can extend beyond the educational setting and positively impact their overall healthcare experiences. Lastly, patient involvement allows for the development of a comprehensive narrative. By sharing their stories and perspectives, expert patients contribute to a more holistic understanding of healthcare that encompasses both clinical knowledge and lived experiences [23, 24]. This narrative-driven approach can help bridge the gap between medical knowledge and the reality of patient experiences. Overall, involving patients in medical education not only benefits the students but also has significant positive impacts on the patients themselves. It empowers them, enhances their knowledge and understanding, and improves their overall satisfaction with healthcare experiences.

### Refining medical education through expert patient contributions: Aston medical school’s collaborative approach with the silver lining brain injury charity

In response to the growing demand from healthcare policymakers to involve patients in various aspects of medical education, Aston Medical School has implemented EP sessions in the early years of the undergraduate medical curriculum. Expert patients from the Silver Lining Brain Injury Charity have participated in EP sessions at Aston Medical School for the past three years. These sessions involve expert patients speaking to students either on-campus or online, facilitated by Clinical Teaching Fellows (CTFs). During these sessions, expert patients discuss their experiences as patients, covering the psychological, biological, and physiological aspects of their conditions and disease progression. Students are encouraged to ask questions, fostering a deeper understanding of patient experiences. During these sessions, expert patients share their life journeys with students. The sessions have garnered overwhelmingly positive feedback from both students and staff.

Our expert patients have actively participated in helped us better design our sessions. Active feedback from our expert patients has been instrumental in refining the design of our sessions. For example, they expressed a preference for smaller groups to facilitate more interactive discussions, leading us to offer both smaller and larger group sessions, tailored to the nature of the session. To accommodate mobility and infection concerns, we have also introduced virtual sessions via platforms such as Zoom and Teams. Fatigue is a constant issue for brain injury survivors and requiring them to repeat their stories several times can become both tiring and boring for them. In response to our expert patient feedback regarding fatigue, we are developing sessions that include pre-recorded patient stories to reduce the need for repetitive storytelling. Additionally, our expert patients emphasised the importance of clearly defining session formats—covering elements such as duration, start times, audience size, facilitation methods, and Q&A protocols—to manage expectations and improve session efficiency. They also suggested enhancing the effectiveness of Q&A sessions by ensuring students have a clear focus for their questions. By establishing a shared understanding of session objectives between expert patients and students, we can foster better interaction and more meaningful learning outcomes.

### Balancing the benefits and challenges of expert patient sessions: Addressing emotional, logistical, and financial concerns in medical education

Educators and students agree on the valuable role of patients as teachers, but there are worries about potential negative impacts. Students and patients may face emotional challenges when dealing with traumatic health narratives, necessitating adequate support and guidance such as providing pre-briefing before the session [18]. Maintaining the quality of patient-led teaching throughout the course is crucial to avoid diminishing the impact of their stories as they become more ‘professionalised’ [18, 28]. Integrating patient-led teaching into the overall course objectives is essential for maximising its effectiveness, as ad hoc sessions may not fully achieve the desired impact [11, 29]. Addressing these concerns is vital to ensure a successful and meaningful patient involvement in medical education.

Multiple reports consistently indicate that educators involved in patient-teacher programs are highly satisfied with the outcomes [18]. Educators believe that these programs enable students to access valuable learning opportunities, gain essential communication skills, and develop a deeper understanding of important patient issues [18]. Educators appreciate the enriching experience that patient-teacher programs provide for students [18]. By interacting with patients, students are exposed to real-life scenarios and challenges, allowing them to apply their theoretical knowledge in practical settings [30]. Interestingly faculty from one of the studies that used expert patients in an interprofessional Health Mentors Programme identified four key crucial factors, which could also be applicable in other patient-led Interprofessional Education (IPE) initiatives [31]. Firstly, in their person-centred learning approach, the faculty members facilitated direct learning between students and patient mentors by providing broad objectives and responding to journals [31]. These journals were personal reflections of students considering what they learned, surprises, insights, assumptions, values, beliefs and further questions. Secondly, they sustained on-going partnerships with community organisations which was vital for mentor recruitment [31]. Thirdly, they embraced minimal instructions and encouraged participants to embrace uncertainty, take responsibility, and nurture creativity [31]. Lastly, the flexibility of integrating the programme into existing courses and adapting delivery to overcome scheduling issues has contributed to their achievements [31].

Many initiatives described in the literature are often limited to isolated educational experiences. To truly promote partnerships between healthcare providers and patients as the foundation of healthcare, there is a need to shift from these isolated initiatives towards coordinated and sustained programs that develop comprehensive patient involvement curricula and foster authentic partnerships at an institutional level [32]. To establish effective partnerships with patients, it may be necessary to implement processes such as facilitated dialogue [33]. This approach can help overcome stereotyping and address the inherent power differential that exists between health professionals and patients, particularly in higher academic settings. Facilitated dialogue creates a safe space for open and honest communication, encouraging active participation from patients and ensuring that their perspectives are valued and incorporated into the curriculum and educational activities [34]. A recent study has underscored the importance of developing training programs to support expert patients in medical education [35]. In this study, researchers created a storytelling program to help patients prepare their narratives in a way that will resonate with the audience [35]. The study reports that patients involved has found the course very helpful. While creating such pilot workshops is crucial, it is essential to ensure that patients are ready to share their stories to minimise the risk of harm and to optimise the experience for students. Although these workshops might identify patients who are not yet ready to be storytellers, they can also be triggering, potentially causing harm simply by attending. Another study developed a self-reflective tool in the form of a questionnaire to assess patients’ readiness to share their stories publicly [36]. Establishing this readiness serves to protect prospective storytellers from unnecessary harm and fosters a more impactful and collaborative partnership between patients and healthcare providers.

The significance of expert patient sessions is recognised by educators; however, several authors have pointed out concerns. Some faculty educators worry that patient stories could be profoundly distressing, necessitating support or debriefings for students to cope with the emotional impact [18, 37]. Another study pointed out the potential reduction in the patient experience’s impact if the same patient participates in the program too frequently [18]. Furthermore, medical educators have raised apprehensions about the substantial costs associated with patient involvement in terms of financial investment and staff time [38]. While one article suggested patient-led teaching as a cost-effective approach compared to physician-led teaching [39], no comprehensive economic evaluation or cost-effectiveness analysis was provided in any of the papers.

### Assessment of long-term benefits of patient interaction

Most of the studies reported on patient involvement in medical education show preliminary data such as student satisfaction questionnaire at the time of session as their evaluation. Most of these sessions are a one-off event, rather than a consistent theme running throughout the curriculum. Studies reporting long term benefits or sustainability are missing in literature. Patient involvement has demonstrated its capacity to enhance crucial attributes such as compassion and emotional intelligence among students in medical education [40]. However, a significant hurdle that lies ahead is the development of assessment techniques that are both valid and reliable, capable of effectively evaluating the multidimensional nature of compassionate empathy which is the highest level of empathy compared to other two (cognitive and emotional empathy) [41]. Assessing this in medical education encompasses a range of dimensions that extend beyond traditional quantitative measures [42]. The challenge lies in capturing the nuanced aspects of compassionate empathy, including understanding patients’ perspectives, demonstrating compassionate care, and effectively communicating and connecting with individuals from diverse backgrounds. To address this challenge, it is essential to devote concerted efforts towards the development and refinement of assessment tools specifically designed to evaluate the multidimensional nature of compassionate empathy [42–44]. These tools should encompass both qualitative and quantitative measures, considering self-assessment, patient feedback, peer evaluations, standardised scenarios, role-playing exercises, and objective structured clinical examinations (OSCEs).

### Reporting expert patient involvement initiatives

The extent to which patients are involved in medical education can vary greatly, ranging from minimal engagement (patient as just part of a paper-based, electronic or web-based case or scenario) to full partnership (sustained patient involvement in education, evaluation and curriculum development at institutional level) [17, 25]. This wide range of involvement poses a challenge when attempting to classify these variables within published works. The apparent lack of an agreed taxonomy makes it difficult to compare studies and creates inconsistencies in the scope of review articles. To address this issue, various classifications, such as the Cambridge framework [26], ladder of involvement [27], and spectrum of involvement [17], have been developed to aid in categorising these levels of patient engagement. Ensuring the consistent utilisation of these classifications is imperative to effectively identify and compare similar initiatives in the field of medical education. By establishing a common framework, researchers and educators can enhance their ability to analyse and evaluate the impact of patient involvement across different educational settings.

In recent years, there has been a notable rise in the number of publications investigating patient involvement in medical education. However, a concerning trend is the lack of comprehensive reporting on critical aspects such as learning outcomes, content, training methods, and other key elements that are essential for effective dissemination and replication of patient involvement initiatives [45]. For future studies in this field, it is crucial to have a solid foundation of clear and relevant theoretical frameworks that inform the design and implementation of patient involvement interventions [25]. This is particularly important when reporting on the relatively unadopted involvement of patients in curriculum design. These studies should be guided by appropriate pedagogical principles to ensure the effectiveness of involving patients in the design of their involvement. Moreover, reporting of research findings and methodologies should be conducted in a manner that supports evidence-based replication and dissemination [46]. Transparent reporting facilitates the sharing of best practices, enables others to replicate successful initiatives, and contributes to the overall advancement of patient involvement in medical education. By addressing these gaps and improving reporting practices, the medical education community can enhance the evidence base and knowledge surrounding patient involvement, leading to wider adoption and implementation of effective strategies across diverse educational settings [47].

## Conclusion

In conclusion, the integration of expert patients into early medical education represents a transformative shift towards a more compassionate and person-centred healthcare system. Expert patients, individuals with firsthand experience and deep understanding of specific health conditions, provide invaluable insights that bridge the gap between theoretical knowledge and practical, compassionate care. The feedback from expert patients highlights numerous benefits, including increased self-confidence, personal satisfaction, and a better understanding of their own health conditions. These sessions not only empower patients but also enhance students’ communication skills, empathy, and understanding of patient perspectives, ultimately shaping more compassionate and effective future healthcare professionals. Expert patients’ suggestions for improving these sessions, such as smaller group settings, virtual participation, pre-recorded stories, and clearer session structures, are critical for optimising the effectiveness of patient involvement. Despite these benefits, challenges remain, including inconsistent terminology, lack of long-term assessment, and the need for comprehensive reporting and coordinated programs. Moving towards sustained and coordinated programs of patient involvement in the design of their own participation requires a systematic approach. Institutions need to prioritise the development and implementation of patient involvement curricula that span the entire education and training process. This includes integrating patient perspectives into various aspects of the curriculum, such as teaching, assessment, and evaluation. This might also involve connecting case studies, patients, simulation suites, and virtual reality to craft life-like clinical scenarios and medical encounters, enhancing the learning experience for medical students [41, 42]. Furthermore, establishing authentic partnerships with patients requires ongoing engagement and collaboration [5]. Institutions should actively involve patients in the design, implementation, and evaluation of educational programs. This collaborative approach ensures that patient voices are heard and respected, enabling the co-creation of educational experiences that truly reflect patient needs and perspectives. By transitioning from isolated initiatives to coordinated and sustained programs, medical education can foster meaningful partnerships with patients, promoting person-centred care and enhancing the overall quality of healthcare. This shift requires a commitment to developing patient involvement curricula and establishing authentic partnerships, supported by processes like facilitated dialogue, that address power dynamics and facilitate open communication.

## Data Availability

No datasets were generated or analysed during the current study.

## Abbreviations

EP session: Expert Patient session
CTF: Clinical Teaching Fellow
IPE: Interprofessional Education
OSCE: Objective Structured Clinical Examinations
